# Involving Persons with Dementia in Social Research: A Literature Review

**DOI:** 10.1093/geroni/igab046.2430

**Published:** 2021-12-17

**Authors:** Pamela Manley, Candace Kemp

**Affiliations:** 1 Georgia State University/Gerontology Institute, Atlanta, Georgia, United States; 2 Gerontology Institute, Georgia State University, Atlanta, Georgia, United States

## Abstract

Abstract Studies involving persons with dementia as research participants have increased over the years, due in part to an emphasis on patient and public involvement in health care and participatory action research. Recent studies indicate a growing trend toward engaging persons with dementia, not only as participants, but also as co-researchers. Further, studies involving persons with dementia as co-researchers and advisers have garnered increased attention due to the inclusion of this population’s unique perspectives and lived experiences. Theoretically, frameworks such as person-centered- and relationship-centered care, also influence and shape the research process. This literature review examines empirical research conducted over the past decade that reports the involvement of persons with dementia as research participants (“research on”) and co-researchers and advisers (“research with”). Among the 27 articles identified, 12 reported “research on,” and 15 reported “research with” persons with dementia. “Research on” targeted participants’ emotional expressions/responses, engagement in exercise/activities; social environment influences, and cognitive training; whereas “research with,” which was mostly qualitative, focused on co-researchers’ perspectives of and experiences with the research process; needs, priorities, and recommendations in research planning; partnering with persons with dementia on the design and development of research instruments, and the importance of critically evaluating the research process. Findings documenting research challenges, complexities, and ethical concerns are also discussed. Overall, findings demonstrate the feasibility of involving persons with dementia in a meaningful way and further affirms that including them as co-researchers is not only beneficial, but has the potential to enhance the entire research process.

